# Outcome of Gastric Bypass Surgery on Patients with Polycystic Ovary Syndrome: A Review

**DOI:** 10.3390/jcm12123940

**Published:** 2023-06-09

**Authors:** Stefan Ghobrial, Johannes Ott, Johanna Steininger, Didier Dewailly, Gerhard Prager

**Affiliations:** 1 Clinical Division of Gynecologic Endocrinology and Reproductive Medicine, Medical University of Vienna, 1090 Vienna, Austria; 2 Faculty of Medicine, University of Lille, 59000 Lille, France; 3 Department of Surgery, Division of General Surgery, Medical University of Vienna, 1090 Vienna, Austria

**Keywords:** gastric surgery, polycystic ovary syndrome, outcome

## Abstract

Polycystic ovary syndrome (PCOS), the most common endocrine disorder in women of reproductive age, is associated with obesity. The most effective method to achieve and maintain long-term weight loss is by the Roux-en-Y gastric bypass (RYGB). In this review, an overview about metabolic and PCOS-specific outcomes after RYGB in obese PCOS women is provided. The RYGB leads to an adequate excess weight loss and reduction in BMI in this patient population. Testosterone levels decline significantly at 6- and 12-months follow-up, as does the incidence of hirsutism and cycle irregularities. Data about fertility are scarce in this patient population. In conclusion, RYGB surgery seems to be an efficient treatment option for obese PCOS patients and leads to weight loss and improvements in metabolic parameters as well as in an improvement of PCOS-specific characteristics. However, larger prospective studies are warranted, which include all PCOS-specific outcome data in one patient population at the same time.

## 1. Introduction

Polycystic ovary syndrome (PCOS) is the most common endocrine disorder in women of reproductive age, with a prevalence of 6–15%. It is diagnosed according to the Rotterdam criteria by the presence of at least two of the following three characteristics: oligo-/anovulation, clinical or biochemical hyperandrogenism, and the presence of polycystic ovaries [1]. It is a common cause of gynecological problems, such as subfertility and abnormal menstrual cycles. Women with PCOS often face other major health concerns as well, including obesity, type 2 diabetes, cardiovascular disease, sleep apnea, psychological disorders, endometrial hyperplasia and even endometrial cancer [2]. It has been shown that women with PCOS are about three times more likely to develop endometrial cancer compared to those without the disease [3], with the risk being even higher in obese women [4]. Notably, obesity (body mass index, BMI > 30 kg/m^2^) is a common trait among women with PCOS [5]. It affects between 30–70% depending on the study settings and the ethnic background of the subjects [6]. A higher body mass index has been found to be a causal factor for developing PCOS, but having PCOS does not seem to have a significant effect on the BMI [7]. Additionally, insulin resistance, type 2 diabetes and cardiovascular disease seem to occur more frequently in obese women with PCOS compared to normal-weight PCOS women [8,9]. Therefore, managing obesity is an important measure for treating PCOS [10].

The most effective method to achieve and maintain long-term weight loss is by Bariatric/Metabolic surgery (BMS). Meta-analyses have shown that surgical interventions, regardless of the type of procedure, lead to greater improvements in weight loss and weight-related comorbidities compared to non-surgical interventions [11]. BMS can be considered for people with severe or morbid obesity (BMI > 40 kg/m^2^) or for those with a BMI under 40 and obesity-related diseases, such as diabetes or insulin resistance. There are few contraindications to BMS, such as poor myocardial reserve, significant respiratory dysfunction, noncompliance with medical treatment and mental disorders of significant magnitude. Common BMS procedures are the Roux-en-Y gastric bypass, adjustable gastric banding, biliopancreatic diversion with duodenal switch and sleeve gastrectomy [11]. The Roux-en-Y gastric bypass (RYGB) is the second most commonly performed bariatric procedure worldwide and is considered to be the gold standard [12,13]. While complications associated with RYGB are not common, there are still some potential complications, such as junction leaks, acute gastric dilatation, delayed gastric emptying, vomiting, wound hernias and intestinal obstruction, which are worrying for some patients. Nutritional deficiencies, such as deficiencies in calcium, vitamin D, vitamin B12, and iron, may also occur and require monitoring and supplementation [11].

Since RYGB surgery is the most effective BMS, this review aims to provide a comprehensive and up-to-date overview of the efficacy and safety of RYGB surgery in the treatment of PCOS and its impact on associated metabolic and reproductive outcomes.

## 2. Materials and Methods

### 2.1. Study Eligibility Criteria

We considered observational retrospective studies, prospective studies and randomized clinical trials for inclusion in this review if they reported quantitative data on Roux-en-Y gastric bypass surgery (RYGB) in women with PCOS, with a minimum of 6 months’ follow-up data.

### 2.2. Literature Search, Study Selection and Data Extraction

We searched the MEDLINE database for published articles. The search was performed in March 2023. No language or date restrictions were applied. Specific Medical Subject Heading (MeSH) terms included “polycystic ovary syndrome” and “gastric bypass”. The reference lists of identified publications were searched manually to identify additional relevant papers. The retrieved titles and abstracts were screened separately by two authors (S.G., J.O.) to identify studies that met the inclusion criteria. The literature search identified 24 references for possible inclusion in this review. The full texts of potentially eligible studies were retrieved and independently assessed for eligibility by the two authors (S.G., J.O.). From these studies, we identified and reviewed eight articles that were relevant and/or addressed the primary research question. Disagreements were resolved by consensus.

A predesigned form was used to collect the following information: name of the first author, publication year, study design, participant characteristics, sample size and outcomes. If outcome data were reported in published figures, DigitizeIt version 2.5 software (I. Bormann, Braunschweig, Germany; https://www.digitizeit.xyz/, accessed on 12 April 2023) was used to reconstruct the data from the publications.

In this analysis, 24 publications were reviewed [14,15,16,17,18,19,20,21,22,23,24,25,26,27,28,29,30,31,32,33,34,35,36,37]. Three articles were excluded because the surgical procedure was specifically not RYGB and another one because the surgical procedure used was not documented [22,23,24,25]. In six other studies, several different bariatric surgical procedures were performed. These studies had to be excluded, since no distinction was made between the surgical methods used when reporting the results [26,27,28,29,30,31]. In two other studies, although all patients underwent RYGB treatment or there was a separate RYGB group, not all patients had PCOS. However, the results of patients with PCOS were not analyzed and evaluated separately, but the results of all RYGB patients were evaluated together, which made it impossible to draw conclusions about the PCOS population [32,33]. Another three studies were excluded because the reported outcomes did not contain any results regarding PCOS [34,35,36], and another one was excluded because neither the article nor the abstract was accessible [37]. Finally, eight of these articles were selected based on appropriate documentation of the outcome data regarding weight loss and comorbidities after RYGB in PCOS patients [14,15,16,17,18,19,20,21]. These include three prospective and five retrospective studies, with a total of 547 patients included in the final review. Notably, there are two studies published by the team of Turkmen et al. [15,17]. After consultation with the authors, it became clear that these studies shared only some patients and were thus both included in the present review.

### 2.3. Outcome Parameters

The focus of this review was on the following outcome parameters: body mass index (BMI, kg/m^2^); body weight (kg); excess weight loss, calculated as: (baseline weight–postoperative weight)/(baseline weight–ideal weight); HbA1c (%; converted if necessary), total testosterone levels (ng/mL); the incidence of impaired glucose tolerance/insulin resistance (IR); the incidence of type 2 diabetes mellitus; and postoperative complications. We chose to present the studies’ data before RYGB (“baseline”) and 6 and 12 months after the operation to provide a more standardized review and ensure better comparability of the data presented. In addition, the following parameters are provided: year of publication; patients’ mean age at the time of surgery; and the number of patients included.

### 2.4. Surgical Procedure

All included patients underwent laparoscopic RYGB using an established technique that has been described previously [13]. In brief, the surgical procedure involves construction of an isolated small gastric pouch of 15–30 cm^3^ size with an antecolic, antegastric Roux limb with a length of 75–150 cm and with stapled end-to-side gastrojejunostomy and side-to-side jejunojejunostomy.

## 3. Results

Detailed overviews on the patient populations and the results of the included eight studies are provided in Table 1 and Table 2. In all reviewed studies, the Rotterdam criteria [1] had been applied for the diagnosis of PCOS. The RYGB surgery had been performed as previously described [12,13].

### 3.1. Weight and Metabolic Outcomes

For better comparability among the studies, follow-up values after 6 and 12 months were selected (Table 1). All in all, the evaluated studies demonstrated that RYGB surgery in obese patients with PCOS led to a significant decrease in BMI (mean baseline levels 44.8–53.4 kg/m^2^, mean levels at 12-months follow-up 29.2–36.4 kg/m^2^), as well as significant improvements in associated comorbidities. These included a decline in Hb1Ac [14,15,18,19] as well as in the incidence of type 2 diabetes mellitus, which was evaluated by three studies (baseline: 10.4–45.8%, 12-months follow-up: 0–5.0%) [19,20,21]. Notably, only three studies reported outcomes after two years [14,19,20]. In two of these, the already achieved weight loss after one year could be maintained [14,20], while there was even more excess weight loss in one report [19].

### 3.2. PCOS-Specific Outcome

Concerning PCOS-specific characteristics (Table 2), a high baseline incidence of hirsutism was found (70.0–95.8%), which decreased to 20.8–50.0% at 12-months follow-up [16,20,21]. Three studies [15,16,17] also focused on total testosterone levels, which declined significantly 6 and 12 months after the operation. Notably, not all studies reported the laboratory methods used, so that the results are only comparable with each other to a limited extent. Menstrual cycle irregularities had been found in up to 100% of included women before the operation [17], and significant improvements up to a rate of 0–44.4% at 12 months follow-up were reported [16,17,20]. Only two studies reported fertility outcomes: Jamal et al. [20] reported 10 patients who were seeking medical attention preoperatively for infertility, defined as the inability of a couple to conceive after one year of trying or the inability to carry a live pregnancy to term. After surgery, four patients no longer desired pregnancy, while the remaining six became pregnant within 3 years of RYGB. These patients revealed a mean excess weight loss of 57%. Of these six patients, five conceived naturally without hormonal therapy and one required intrauterine insemination. In the population of Eid et al. [21], five women suffered from infertility, defined the same as in the other previous study. Postoperatively, all five patients who desired to conceive were able to do so within about two years without clomiphene stimulation.

### 3.3. Surgical Complications

Empirically, one major concern of the patients is the safety of the procedure. Only three of the reviewed studies [14,16,21] reported postoperative complications, and one explicitly reported none. In the population of Jamal et al. [20], there were no postoperative complications (0/20, 0%). In the study of Eid et al. [16], two patients (2/14, 14.3%) experienced a postoperative complication, namely postoperative upper gastrointestinal or intra-abdominal bleeding, with the second patient requiring laparoscopic exploration with clot evacuation. Both patients recovered well from their complications. In the other study published by Eid et al. [21], one patient (1/24, 4.2%) experienced a post-operative gastrointestinal bleeding that resolved without any surgical intervention. Ahmed et al. [14] reported one jejuno-jejunal anastomotic leak that required emergency laparotomy and the subsequent reversal of the jejunostomy a year later (1/30, 3.3%).

None of the studies reported any deaths. Combining the incidence of a postoperative complication in all these populations, the incidence is about 4.5% (4/88).

## 4. Discussion

The reviewed studies provide evidence that RYGB surgery can result in corrective endocrine and metabolic changes in women with PCOS, which may lead to remission of the condition. However, we were able to identify only eight relevant and sound studies according to our criteria, which included a total of 547 PCOS patients with RYGB surgery. Given the fact that the majority of these patients were included in one large retrospective study (*n* = 389) and that the authors did not report data about hirsutism, menstrual cycle irregularities, fertility aspects and testosterone [18], data on PCOS-specific outcomes in this population are considerably scarce.

### 4.1. BMI

However, in the mentioned large study, Gomez-Meade et al. [18] were able to show a significant decrease in BMI after surgery, and this is in line with all other review studies (Table 1) [14,15,16,17,18,19,20,21]. This underlines the well-known efficacy of RYGB surgery for achieving weight loss [11], also in PCOS women. Notably, this effect seemed to exist regardless of ethnicity. Gomez-Meade et al. compared the data of Hispanic, black and white study participants. Nevertheless, ethnic group differences were found in the risk factors for cardiometabolic diseases among women with PCOS, including insulin resistance and dyslipidemia. Although all women demonstrated an improvement in HbA1c, alanine aminotransferase (ALT), total cholesterol, low-density lipoprotein (LDL) and triglycerides, only Hispanic women showed a significant decrease in all of these parameters 12 months postoperatively [18]. It seems worth mentioning that weight loss after RYGB surgery in women with PCOS was comparable to patients without PCOS (14,19). This is in contrast to the study of Dixon and O’Brien [24], where patients underwent laparoscopic gastric banding. It reported a significantly lower excess weight loss over 12 months in women with PCOS, women with a history of gestational diabetes and patients with type 2 diabetes compared to other patients. This might be seen as another indicator of the superiority of RYGB surgery over gastric banding.

### 4.2. PCOS

Notably, Turkmen et al. [17] also examined the changes in eating behavior in nine women with PCOS after RYGB surgery. For this purpose, patients completed a Three-Factor Eating Questionnaire (TFEQ) addressing eating behavior. The scores for uncontrolled eating and emotional eating decreased, whereas the scores for cognitive restraint increased after surgery. When the serum levels of allopregnanolone, a major metabolite of progesterone and very potent modulator of the GABA_A_ receptor, which stimulates food intake and might be involved in PCOS pathogenesis, were determined, the presurgical allopregnanolone levels were significantly correlated with uncontrolled eating. Although this suggests that allopregnanolone might be part of the mechanism underlying the abnormal eating behavior of obese PCOS patients by causing loss of control over food intake, the serum allopregnanolone level and the allopregnanolone/progesterone ratio were unchanged after surgery [17].

Apart from this hypothesis, it could not be shown that RYGB surgery could directly affect PCOS-pathogenesis. However, concerning PCOS-specific outcomes, it could be demonstrated in three studies [15,16,17] that the weight loss achieved by RYGB surgery was accompanied by a relevant decrease in testosterone levels. This is also well-known for other interventions in PCOS women with weight-reducing effects [38,39].

### 4.3. Cycle Irregularities

Another important PCOS-specific topic is cycle irregularities. In the study conducted by Eid et al. [16], ten women with oligomenorrhea prior to surgery were identified, all of whom reported regular monthly cycles post-surgery, suggesting a resumption of ovulation. However, caution is advised when interpreting self-reported menstrual frequency. In a recent study of 29 women who underwent the same surgery, only 48% reported regular periods before the surgery. However, an objective urinary marker of ovulation, pregnanediol 3-glucuronide (Pd3G), showed that 90% of patients had indeed been ovulatory prior to surgery [40,41]. Notably, it has been claimed that the resumption of ovulatory menstrual cycles after RYGB surgery may indicate improvements in metabolic abnormalities, while PCOS patients who remained anovulatory still had metabolic abnormalities. In the study of Turkmen et al., there were significant improvements in the waist circumference, systolic blood pressure, serum allopregnanolone level, and serum progesterone level between PCOS patients who had remained anovulatory and those with a recovered ovarian function [15]. This presumption is supported by the findings of another retrospective survey. Of 195 women who had undergone bariatric surgery, 92 were anovulatory before surgery with menstrual cycles longer than 35 days. After surgery, 71.4% of anovulatory women regained normal menstrual cycles and thus normal ovulation. Those women had lost significantly more weight than those who remained anovulatory (61.4 kg vs. 49.9 kg) [42].

### 4.4. Fertility

Regarding fertility outcomes, only two of the included studies [20,21] examined these. Both studies showed that RYGB intervention alone was sufficient to significantly increase fertility rates in infertile women with PCOS postoperatively. A meta-analysis by Chang et al. [43] comparing bariatric surgery with non-invasive methods, such as metformin, confirmed the clear superiority of surgery. Patients who underwent bariatric surgery were more than twice as likely to become pregnant as those treated with metformin alone (34.9% vs. 17.1%).

### 4.5. Adolescence

However, bariatric surgery appears to be beneficial not only for women of reproductive age, but also for adolescent patients. Miller et al.’s review [44] highlights that research on postoperative outcomes has shown several benefits of bariatric surgery, especially RYGB, such as the resolution of obstructive sleep apnea and significant improvements in hyperinsulinemia and other metabolic complications associated with obesity and PCOS, in adolescent patients [45].

### 4.6. Restricitions

A limiting factor of this review is the restriction of the follow-up period to 12 months. Some studies [14,19,20,21] have reported better results after this period, but we limited the time frame to 12 months to ensure a better comparability of results. Another common challenge reported by most of the studies was the partial lack of compliance during follow-up and thus limited follow-up data.

## 5. Conclusions

The aim of this review was to provide an overview of the efficacy and safety of RYGB surgery in the treatment of PCOS and its impact on associated metabolic and reproductive outcomes. In conclusion, RYGB surgery seems to be an efficient treatment option for obese PCOS patients and leads to weight loss and improvements in metabolic parameters as well as an improvement of PCOS-specific characteristics. However, larger prospective studies are warranted, which include all PCOS-specific outcome data in one patient population at the same time.

## Figures and Tables

**Table 1:** Overview of studies on the use of RYGB surgery in obese PCOS patients: weight and metabolic data.

Ref. Nr.	Year of Publ.	Sample Size	Age (Years)	Time Point	BMI (kg/m^2^)	Body Weight (kg) & Mean Weight Loss (kg)	% Excess Weight Loss	IR (*n*, %)	T2DM (*n*, %)	HbA1c (%)
[14]	2021	30	35.5 ± 1.3	Baseline	53.4 ± 1.7	145.7 ± 5.1	-	-	7 (23.3)	8.78 ± 2.81
				6 months	-	-	-		-	-
				12 months	36.4 ± 0.8	47.5 ± 1.9	62.6 ± 3.3		-	5.66 ± 2.64
[15]	2015	13	29.9 ± 7.1	Baseline	47.2 ± 7.6	-	-	6 (46.2)	1 (7.7)	4.55 ± 0.35
				6 months	35.5 ± 7	-	-	-	-	3.92 ± 0.52
				12 months	-	-	-	-	-	-
[16]	2014	14	36.3 ± 8.4	Baseline	44.8 ± 5.9	110 ± 3.6	-	-	4 (28.6)	-
				6 months	32.4 ± 5.9	37 ± 11	-	-	-	-
				12 months	29.2 ± 5.9	45 ± 13	66.5	-	-	-
[17]	2014	9	31.4 ± 7.4	Baseline	47.2 ± 8.9	-	-	-	-	-
				6 months	35.7 ± 8.0	-	-	-	-	-
				12 months	31.8 ± 9.3	-	-	-	-	-
[18]	2013	389	40.9 ± 12.9	Baseline	45.8 ± 1.1	121.3 ± 3.5	-	-	-	6.24 ± 0.23
				6 months	-	-	-	-	-	-
				12 months	36.1 ± 1.1	25.7 ± 0.7	52.4 ± 2.5	-	-	5.66 ± 0.18
[19]	2013	48	33 ± 7.1	Baseline	50.9 ± 7.0	138.5 ± 20	-	16 (33.3)	5 (10.4)	5.93 ± 1.91
				6 months	-	-	-	-	-	-
				12 months	34.5 ± 0.6	-	-	Total numbers not provided, 11.5%	0	- *
[20]	2011	20	32 ± 5.8	Baseline	52.8 ± 9.1	147.9 ± 24.5	-	-	9 (45.0)	-
				6 months	-	45 ± 6	-	-	3 (15.0)	-
				12 months	34.3 ± 5.7	50 ± 7	52.2	-	1 (5.0)	-
[21]	2005	24	34 ± 9.7	Baseline	50.0 ± 7.5	138.8 ± 20	-	-	11 (45.8)	-
				6 months	-	-	-	-	-	-
				12 months	30.0 ± 4.5	-	56.7 ± 21.2	-	0	-

Abbreviations used: IR, insulin resistance; T2DM, type 2 diabetes mellitus; * after 24 months: 3.23 ± 2.64%.

**Table 2:** Overview of studies on the use of RYGB surgery in obese PCOS patients: PCOS-specific data.

Ref. nr.	Year of Publ.	Sample Size	Age (Years)	Time Point	Hirsutism (*n*, %)	Menstrual Cycle Irregularities	Fertility Aspects	Testosterone (ng/mL)
[14]	2021	30	35.5 ± 1.3	Baseline	-	-	-	-
				6 months	-	-		-
				12 months	-	-		-
[15]	2015	13	29.9 ± 7.1	Baseline	-	13 (100.0)	-	1.85 ± 0.50
				6 months	-	6 (46.2)		1.12 ± 0.38
				12 months	-	-		-
[16]	2014	14	36.3 ± 8.4	Baseline	11 (78.6)	10 (7.14)	-	2.05 ± 0.28
				6 months	-	0		1.08 ± 0.14
				12 months	7 (50.0)	0		1.17 ± 0.15
[17]	2014	9	31.4 ± 7.4	Baseline	-	9 (100.0)	-	2.00 ± 0.70
				6 months	-	4 (44.4)		1.08 ± 0.52
				12 months	-	4 (44.4)		0.99 ± 0.48
[18]	2013	389	40.9 ± 12.9	Baseline	-	-	-	-
				6 months	-	-		-
				12 months	-	-		-
[19]	2013	48	33 ± 7.1	Baseline	-	25 (52.3%)	21 (43.2%) had fertility concerns before the operation; no outcome data provided	-
				6 months	-	-		-
				12 months	-	- *		-
[20]	2011	20	32 ± 5.8	Baseline	14 (70.0)	17 (85.0%)	6/6 conceived within 3 years: 5 without hormonal therapy, 1 with in utero insemination	-
				6 months	11 (55.0)	11 (55.0%)		-
				12 months	9 (45.0)	3 (15.0%)		-
[21]	2005	24	34 ± 9.7	Baseline	23 (95.8)	24 (100.0)	5/5 conceived without clomiphene within about 2 years	-
				6 months	12 (50.0)	0		-
				12 months	5/23 (20.8)	0		-

* after 24 months: 10/48 (20.5%)

## Data Availability

All the data is available within the study. This process can be initiated upon request to the corresponding author.
